# Ptk7 Marks the First Human Developmental EMT *In Vitro*

**DOI:** 10.1371/journal.pone.0050432

**Published:** 2012-11-28

**Authors:** David N. Chan, Soheila F. Azghadi, Jun Feng, William E. Lowry

**Affiliations:** 1 Department of Molecular Cell and Developmental Biology, University of California Los Angeles, Los Angeles, California, United States of America; 2 Eli and Edythe Broad Center for Regenerative Medicine, University of California Los Angeles, Los Angeles, California, United States of America; 3 Jonsson Comprehensive Cancer Center, University of California Los Angeles, Los Angeles, California, United States of America; 4 Molecular Biology Institute, University of California Los Angeles, Los Angeles, California, United States of America; 5 Department of Medical and Molecular Pharmacology, University of California Los Angeles, Los Angeles, California, United States of America; Indian Institute of Toxicology Reserach, India

## Abstract

Epithelial to mesenchymal transitions (EMTs) are thought to be essential to generate diversity of tissues during early fetal development, but these events are essentially impossible to study at the molecular level *in vivo* in humans. The first EMT event that has been described morphologically in human development occurs just prior to generation of the primitive streak. Because human embryonic stem cells (hESCs) and induced pluripotent stem cells (hiPSCs) are thought to most closely resemble cells found in epiblast-stage embryos prior to formation of the primitive streak, we sought to determine whether this first human EMT could be modeled *in vitro* with pluripotent stem cells. The data presented here suggest that generating embryoid bodies from hESCs or hiPSCs drives a procession of EMT events that can be observed within 24–48 hours after EB generation. These structures possess the typical hallmarks of developmental EMTs, and portions also display evidence of primitive streak and mesendoderm. We identify PTK7 as a novel marker of this EMT population, which can also be used to purify these cells for subsequent analyses and identification of novel markers of human development. Gene expression analysis indicated an upregulation of EMT markers and ECM proteins in the PTK7+ population. We also find that cells that undergo this developmental EMT retain developmental plasticity as sorting, dissociation and re-plating reestablishes an epithelial phenotype.

## Introduction

Human pluripotent stem cells (hPSCs) are widely regarded to resemble the epiblast of a developing embryo [1]. It has been demonstrated that embryoid bodies can be routinely generated from both hESCs and hiPSCs [2], [3], [4], [5], [6]. Embryoid bodies (EBs) are cell aggregates generated from pluripotent stem cells, which spontaneously differentiate and have the potential to form all three embryonic germ layers [7]. EBs have been adopted as a standard to assess the pluripotency and differentiation potential of pluripotent stem cells [2], [3]. Understanding how human pluripotent stem cells (hPSCs) spontaneously undergo differentiation is important for: (1) understanding how to maintain pluripotency in culture; (2) improving protocols to induce different progeny cell types of significance; (3) establishing an “*ex vivo*” model of human development.

Because of their three-dimensional organization and cell of origin, it has been suggested that embryoid bodies can be used as a model to study early human development [7], [8]. The early events of development have been well studied in model organisms such as sea urchin, chicken, and mouse [9], but the process in human is still elusive at the molecular level. Although the mouse model has generally been accepted as a close alternative to studying human development, there are well-established differences between murine and human development. First and foremost, mouse embryonic development occurs much faster than human development. Secondly, the molecular events underlining development are not identical [10], [11], [12]. Understanding exactly how human development occurs will be of value to modeling congenital diseases and other developmental defects, particularly with the advent of human induced pluripotent stem cells (hiPSCs), where disease states can be captured *in vitro* [13], [14]. Here we demonstrate that embryoid bodies from either hESCs or hiPSCs can be used to demonstrate early human developmental events such as epithelial-to-mesenchymal transition (EMT).

EMT is critical for the development of many cell types during normal development of all species [15]. EMT is known to underlie several important developmental events, such as gastrulation and neural crest migration [15], [16], [17]. The first wave of primary EMT after implantation occurs when mesendodermal progenitors are formed from the developing epiblast, prior to the formation of the primitive streak [15]. Here we show that a spontaneous EMT event occurs in human pluripotent stem cell (hPSC, used here to indicate both hESC and hiPSC) culture as well as in EBs. In addition to a three-dimensional model for development, the use of EBs (with serial sections) enabled the identification of multiple markers in the same population that underwent EMT. Since hPSC are thought to most closely resemble cells from the developing epiblast [1], we speculate that the EMT we observed in hPSCs and EBs could correlate with this step of gastrulation in human development.

Protein tyrosine kinase 7 (PTK7) is a transmembrane receptor initially identified in colon carcinoma as colon carcinoma kinase-4, CCK4 [18]. Orthologs of PTK7 have been identified in Hydra (*Lemon*), *Drosophila* (*offtrack, otk*), chicken (*kinase-like gene, KLG*) and mouse (*Ptk7*) [19]. PTK7 has been shown to play critical roles in developmental processes, most notably the regulation of gastrulation and neural tube closure [20], [21]. Both of these events entail convergent extension, which involves the regulation of planar cell polarity [22], [23], in which PTK7 is a crucial regulator [21], [24], [25]. In addition, PTK7 is also involved in other developmental events such as axon guidance, neural crest migration and cardiac morphogenesis [26], [27], [28], [29], [30].

EBs have been utilized to isolate populations that have undergone EMT to become the gastrula organizer or mesodermal progenitor [8], [31], however, there are no clear methods to isolate or capture the initial EMT population and study them at the molecular level. Because of its important role in early development, we investigated the expression of PTK7 in embryoid body development. We found PTK7 to be a reliable, cell surface marker for EMT in EBs. To further define these cells, we compared them to definitive endoderm derived from hPSCs. The characterization of these stages of human development can potentially unveil novel markers that are important for human development, and provide a platform for the study of early embryonic developmental disorders.

## Materials and Methods

### Cell Culture

Pluripotent stem cells on feeders were cultured as described [6], [32] with UCLA Embryonic Stem Cell Committee Oversight (Escro) approval. Briefly, Pluripotent stem cells on feeders were maintained in DMEM/F12 supplemented with L-glutamine, nonessential amino acids, penicillin–streptomycin, knockout serum replacement (Invitrogen), and 10 ng/ml basic FGF (R&D Systems) and passaged with collagenase (1 mg/ml Type IV Collagenase, GIBCO). Feeder-free pluripotent stem cells were maintained with mTeSR1 (Stem Cell Technologies) and passaged mechanically (using 200 uL pipet tips or StemPro EZPassage Tool, Invitrogen) or by collagenase (1 mg/ml Type IV Collagenase, GIBCO). Spontaneous differentiation of pluripotent stem cells were induced using NutriStem media (Stemgent) without FGF supplement. Neural rosette derivation and NPC purification were performed as described. Briefly, pluripotent stem cells or cell-reaggregates were treated for at least 7 days with DMEM/F12 supplmented with N2 and B27 supplements (1X, Invitrogen), 20 ng/mL basic FGF (R&D systems), RA (1 µM, Sigma) and SAG (1 µM, Calbiochem). Derivation of definitive endoderm was performed as described. Briefly, pluripotent stem cells or cell re-aggregates were treated with DMEM/F12 containing 100 ng/mL ActivinA (Peprotech) for 3 days, with 0% FBS at Day0, 0.2% FBS at Day1, and 2% FBS at Day2.

### Generation of Embryoid Bodies

Embryoid Bodies were generated using established methods [6]. Briefly, embryonic stem cell colonies were dissociated from the plate mechanically (using 200 uL pipet tips or StemPro EZPassage Tool, Invitrogen) or by collagenase (1 mg/ml Type IV Collagenase, GIBCO). The cell colonies were centrifuged at 200 g, 5 minutes and then gently pippeted onto ultra-low attachment plates (Corning). Embryoid bodies were kept in NutriStem Media (Stemgent) without FGF supplements for 0 to 10 days.

### Serial Sections of Embryoid Bodies

Embryoid bodies were embedded in O.C.T. Compound (Sakura) in standard cryomolds (Sakura), rapidly frozen on dry ice, and stored at −80 for at least 30 minutes. The frozen embryoid bodies were sectioned on a CM3050S cryostat (Leica) at 6–10 uM thickness. Only serial sections were numbered and employed for subsequent analyses.

### Immunofluorescence

Immunostaining was performed as described [32]. Embryoid body sections or cells on coverslips were fixed in 4% PFA, blocked for 5 to 30 min, stained with primary antibody overnight at 4°C, washed 3X with PBST, incubated with secondary antibody at RT for 1 hr, washed 3X with PBST, mounted with Prolong Gold (Invitrogen) and imaged. All imaging was performed on Zeiss Axio Imager A1. Primary Antibodies used include the following: chicken anti-Vimentin (Covance, 1∶1000), mouse anti-PTK7 (Sigma-Aldrich, 1∶100), mouse anti-NESTIN (Covance, 1∶1000), mouse anti-SOX17 (R&D Systems, 1∶50), rabbit anti-EOMES (Abcam, 1∶100), rabbit anti-HMGA2 (Santa Cruz, 1∶100), mouse anti-EPCAM, rabbit anti-cleaved CASPASE3, rabbit anti-ECADHERIN, rabbit anti-LIN28A, rabbit anti-LIN28B, rabbit anti-NANOG, rabbit anti-OCT4, rabbit anti-SOX2, rabbit anti-SLUG (Cell Signaling Technology, all at 1∶100), rabbit anti-NCADHERIN (Santa Cruz, 1∶100), rabbit anti-Ki67 (Abcam. 1∶600) and goat anti-SOX1 (1∶200). Secondary antibodies used include the following: DyLight 488, 649 Donkey anti-chicken IgG, DyLight 649 Donkey anti-goat IgG (Jackson Immunoresearch, 1∶300); AlexaFlour 488, 647 Donkey anti-mouse IgG, AlexaFlour 488, 568 Donkey anti-rabbit IgG (Invitrogen, 1∶500).

### FACS-Isolation of Embryoid Bodies and Embryonic Stem Cells

Embryoid bodies were dissociated with TrypLE and then filtered through a 40 µm cell strainer (BD Biosciences). Embryonic stem cells were dissociated with PBS and filtered through a 40 µm cell strainer. Dissociated cells were blocked with 5% FBS in PBS (FACS buffer), stained with primary antibody for 30 min, washed 2X with FACS buffer, incubated with secondary antibody for 30 min, washed 3X with FACS buffer, stained with 7AAD for 10 min, then filtered through 40 um cell strainer before FACS. FAC-sorting was performed at the UCLA BSCRC FACS Core with FACSAria II (BD Biosciences).

### Cell Re-aggregation from FACS Isolates

FACS-captured populations were re-aggregated following previously established protocols [31]. Briefly, 15000 cells were resuspended into each well of a U-shaped 96-well low-cell binding plates (Nunc) in the presence of Nutristem media (Stemgent) supplemented with 10 ng/mL of basic FGF (R&D systems), 10 uM Rock inhibitor (Y27632, Tocris) and 5 ug/mL fibronectin (BD Biosciences), then centrifuged (Beckman Coulter Allegra X-15R) at 1000 rpm for 5 min at room temperature, then returned to incubator. After 16 hrs, Re-aggregates were carefully pipepted from the 96-well plate onto tissue culture plates coated with Matirgel (BD Biosciences).

### Expression Analysis

RNA isolation was performed with either Stratagene Absolutely RNA miniprep kit or Qiagen RNEasy micro kit; reverse transcription, and real-time PCR were performed as described [6], [32]. Microarray profiling was performed with Affymetrix Human HG-U133 2.0 Plus arrays as described [6], [32]. Data were normalized with Robust Multichip Algorithm in Genespring. Probe sets that were not expressed at a raw value of >50 in at least 10% of samples were eliminated from further analysis.

## Results

### Establishing the Embryoid Body as a Model System for Studying Early Human Fate Decisions

**Figure 1:**
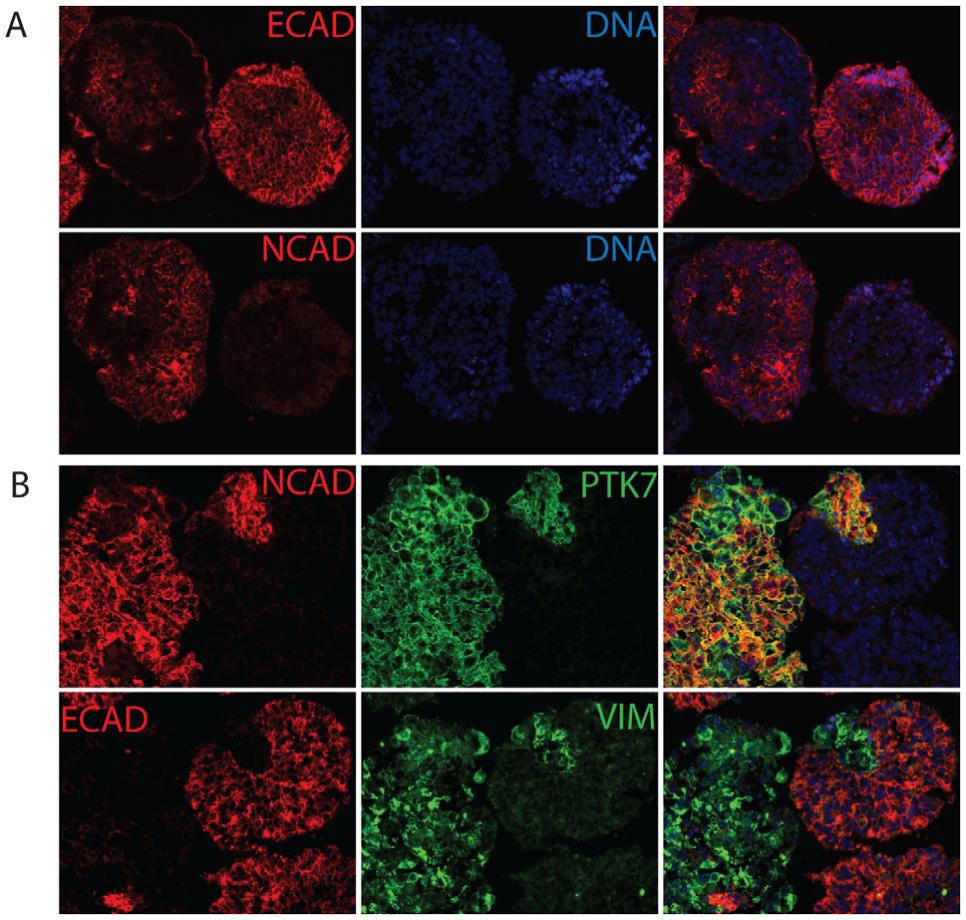
PTK7+ population displays mesenchymal markers but not epithelial markers. (A) Epithelial and mesenchymal markers were mutually exclusive in hEBs. Immunofluorescence staining of H9 hEB serial sections. Serial sections of H9 Day5 hEBs were stained with epithelial markers (E-CADHERIN) and mesenchymal markers (N-CADHERIN). (B), PTK7+ population displayed mesenchymal markers but not epithelial markers. Immunofluorescence staining of H9 hEB serial sections. Serial sections of H9 24 hr hEBs were stained with PTK7 (green), epithelial marker (E-CADHERIN, red) and mesenchymal markers (N-CADHERIN, red; VIMENTIN, green). The rightmost column shows the merged images from two fluorescent channels and DAPI. All images taken at 20X.

To demonstrate the utility of embryoid bodies as model system, we investigated embryoid bodies that were differentiated for 5 days. Immunofluorescence of serial EB cyro-sections revealed that E-CADHERIN (E-CAD) and N-CADHERIN (N-CAD), markers for epithelial and mesenchymal cells respectively, were mutually exclusive (Fig. 1A). EBs at earlier stages of differentiation also displayed the same mutual exclusivity between E-CAD and N-CAD (Fig. 1B and data not shown). Since undifferentiated hESCs/hiPSCs express E-CAD but not N-CAD, this suggested that the N-CAD positive cells represented a switch from an epithelial to a mesenchymal state. In mammalian development, the first EMT occurs during gastrulation, therefore we examined whether these N-CAD positive cells co-expressed other markers relevant to gastrulation. PTK7 is a transmembrane protein that is critical for normal gastrulation in mouse and zebrafish [20], [21]. In serial sections of EBs, PTK7 was shown to be co-expressed in ECAD−/NCAD+ cells. In addition, PTK7 positive cells also displayed upregulation of VIMENTIN (VIM), another marker for mesenchymal cells (Fig. 1B). Interestingly, these cell fate decisions did not appear in a random fashion in dispersed cells throughout the EB, but instead occurred in groups of cells, reminiscent of coordinated cell fate specification. We studied the pattern of PTK7 co-expression with epithelial and mesenchymal markers and determined that from Day0 to Day4, PTK7 faithfully corresponded to a population that co-expressed mesenchymal marker (N-CAD) but was devoid of epithelial marker (E-CAD). After 5 days or more of differentiation, almost all cells became PTK7 positive and the specificity of PTK7 in demarking a non-epithelial population dissipated (Fig. S1, S2, S3). We therefore focused on embryoid bodies in suspension culture for 24–48 hrs.

### PTK7 is Expressed in Populations that have Undergone Epithelial-to-mesenchymal Transition

**Figure 2:**
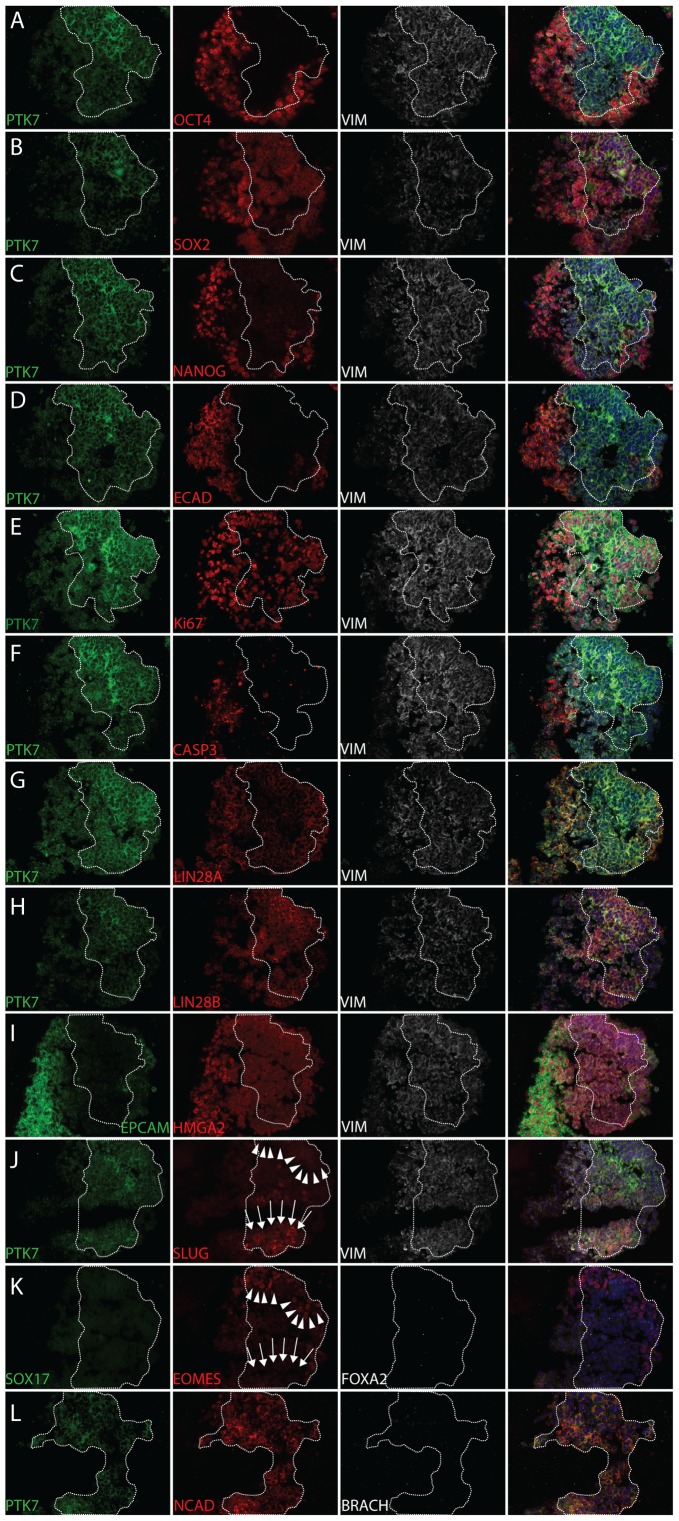
Pluripotency, lineage, and viability marker analysis of PTK7 population in hEBs. Immunofluorescence staining of XFiPSC2 hEB serial sections. White boundary indicates the PTK7 population. In cases where there were no PTK7 staining in a particular section, the boundary was extrapolated from adjacent sections. XFiPSC2 hEBs were cultured for 24 hrs before cryosectioned. Serial sections of XFiPSC2 24 hr hEBs were stained with PTK7 (green), epithelial markers (E-CADHERIN, red; EPCAM, green), mesenchymal markers (N-CADHERIN, red; VIMENTIN, white), pluripotency markers (OCT4, SOX2, NANOG, red), developmental genes (LIN28A, LIN28B, HMGA2, red), primitive streak markers (SLUG, red (arrows); Brachyury, white) and endodermal markers (SOX17, green; EOMES (arrowheads), red; FOXA2, white). The 4^th^ column shows the merged images from three fluorescent channels and DAPI. All images taken at 20X.

EBs were generated from hESC (HSF1, H9) [2], [33] and hiPSC (hiPSC2, XFiPSC2) [6], [34] lines (with or without feeders) under standard culture conditions. EBs were embedded and cryo-sectioned before immunofluoresence staining. Serial sections were stained and analyzed together to display expression of pluripotency and EMT markers. We observed that most PTK7 positive cells in EBs displayed a loss of pluripotency markers such as OCT4 and NANOG and a loss of epithelial markers such as E-CAD and EPCAM (Fig. 2A,C,D,I and Fig. S4A,C,D,I). On the other hand, PTK7 positive populations strongly expressed mesenchymal markers such as N-CAD and VIM (Fig. 2 all, Fig. S4 all and data not shown). Unexpectedly, OCT4 and SOX2 were still expressed in some PTK7 positive clusters (Fig. 2B and Fig. S4A,B), indicating that this EMT was likely a very early differentiation event.

To assay for proliferation and viability, the PTK7-positive populations were also co-stained with Ki67 (for proliferation) and cleaved CASPASE3 (for apoptosis). PTK7-positive populations were largely proliferating and few cells underwent apoptosis (Fig. 2E,F, S4E,F and S5). We also assayed for markers for developmental potential such as LIN28A, LIN28B, and HMGA2. LIN28 protein expression correlates with developmental maturity, whereas HMGA2 can facilitate EMT [35]. While HMGA2 appeared unchanged (Fig. 2I and Fig. S4I), there appeared to be a subtle switch from LIN28A to LIN28B when some cells started expressing PTK7 and underwent EMT differentiation (Fig. 2G,H). To determine whether these cells were capable of both mesodermal and endodermal differentiation, we characterized mesendodermal markers such as SLUG and EOMES. In all stainings analyzed, SLUG and EOMES-expressing cells were strictly within PTK7-positive populations (Fig. 2J and K, Fig. S4J and data not shown). Of note, SLUG and EOMES were expressed in a mutually exclusive fashion (Fig. 2J and K, note arrowheads and arrows), suggesting possibly different mesendodermal outcomes in individual clusters within the PTK7 positive population. We did not see expression of markers for lineage commitment such as BRACHYURY (mesoderm), SOX17 and FOXA2 (endoderm), indicating the lack of lineage complete commitment at this point (Fig. 2K and L, Fig. S4K and L). This is in stark contrast with hPSCs treated with Activin A over a similar timecourse that uniformly adopted an endodermal fate (Fig. S8). Importantly, these observations were made consistently across both hESC and hiPSC lines, and in cultures with and without feeders. The robustness of this EMT suggests that this process is intrinsic to EBs and not subject to subtle variance of culture conditions, potentially allowing the application of this method universally.

### PTK7 Expression Precedes the Epithelial-to-mesenchymal Transition

**Figure 3:**
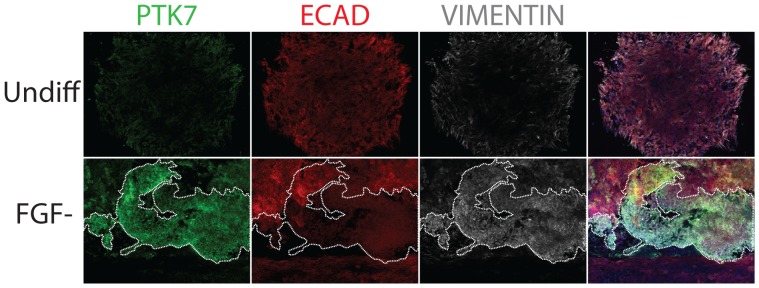
PTK7 marked cells that underwent EMT in adherent differentiation of hPSCs. Immunofluorescence staining of adherent XFiPSC2. White boundary indicates the PTK7 population. XFiPSC2 were plated on coverslips and fixed before immunostaining with PTK7 (green), epithelial maker (E-CADHERIN, red) and mesenchymal marker (VIMENTIN, white). Top row: undifferentiated XFiPSC2. Bottom row: XFiPSC2 differentiated in low FGF conditions for 2 days. The 4^th^ column shows the merged images from three fluorescent channels and DAPI. All images taken at 10X.

To understand whether this EMT can occur on hPSC cultures in the absence of EB formation, we allowed adherent hESC and hiPSC to undergo differentiation *in situ* over 2 to 5 days by culturing in low FGF2 conditions, and performed immunofluoresence staining on fixed colonies. Undifferentiated hESC/hiPSC colonies expressed E-CAD and had no PTK7 staining (Fig. 3, top row). Although the majority of PTK-expressing cells in differentiating cultures were depleted of E-CAD and other pluripotent markers, a small population of which still expressed E-CAD (Fig. 3, bottom row). This suggested that PTK7 expression preceded loss of E-CAD and that the full EMT event might require the physical reorganization imparted by EB formation.

We speculate that simply the rearrangement of cells from 2-D cell culture into a sphere and the subsequent shape changes induced in such an event might drive cell fate decisions in this case, as has been proposed in other settings [36], [37]. To further define the molecular basis for this EMT event and to better understand the consequences, we isolated both PTK7 positive and PTK7 negative populations and performed gene expression profiling.

### Capture and Transcriptional Profile of PTK7+ Population

**Figure 4:**
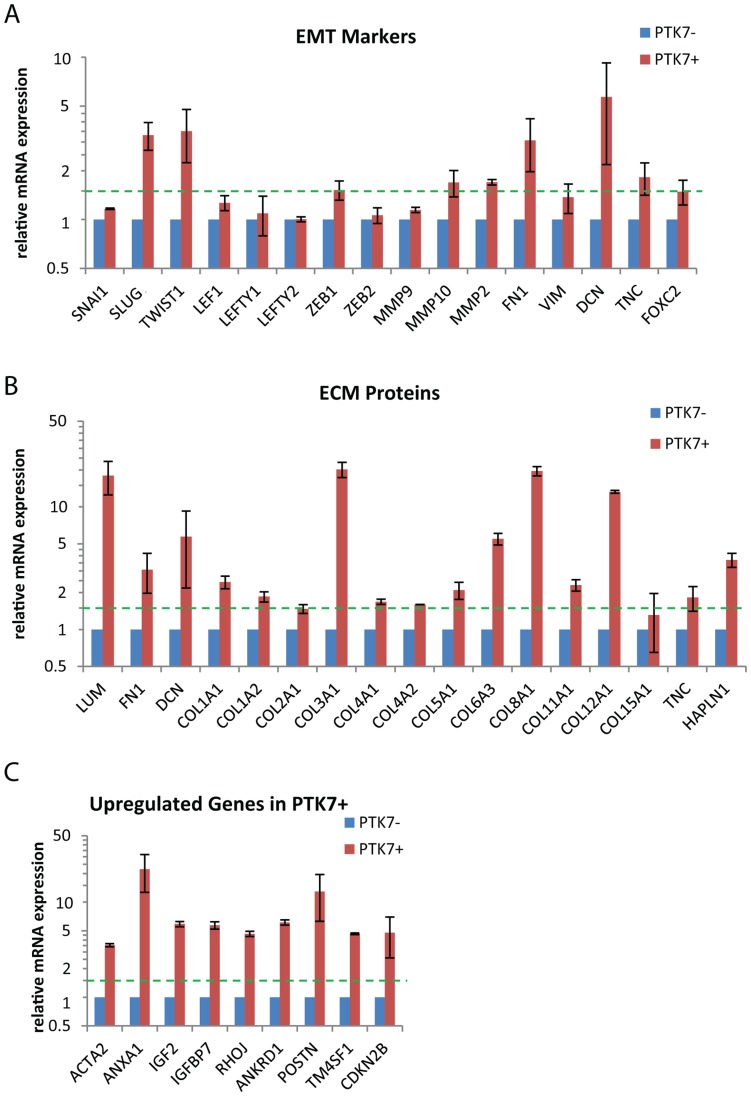
EMT marker and ECM protein analysis on undifferentiated hPSC, PTK7−, PTK7+ and DE populations. Feeder-free H9 and XFiPSC2 (differentiated for 2 days) were sorted into PTK7+ and PTK7**−** populations. Expression level was determined by microarray analysis as described in Materials and Methods. All samples were normalized to PTK7**−** from the same sort. Shown are the averages of normalized readings for PTK7+ and PTK7**−**, with standard error across two experiments. The relative expression of all samples was analyzed for (A) EMT markers, (B) ECM proteins and (C) genes that are upregulated in PTK7+ samples.

The antibody used to identify PTK7 expression also appeared suitable to isolate cells by FACS. Therefore, we sorted PTK7 positive and negative populations from differentiating hPSC culture (H9 and XFiPSC2) to perform transcriptome profiling. We first validated the sorting strategy by RT-PCR as shown in Fig. S6. From microarray analysis, the PTK7 positive population displayed upregulation of markers of EMT and gastrulation, such as *TWIST, SLUG, ZEB1, MMP2, MMP10, FOXC2* and *DECORIN* (Fig. 4A). Fibronectin (*FN1*) and fibronectin-interacting proteins, including *DECORIN, LUMICAN, TENASIN-C* and a panel of collagens (*COL1A1, COL2A1, COL3A1, COL4A1, COL4A2, COL5A1, COL6A1, COL6A2, COL6A3, COL8A1, COL11A1, COL12A1*), were also significantly induced (Fig. 4B). An intact fibronectin matrix complex is essential for developmental patterning and gastrulation [38], [39], while DECORIN has been shown to play a role in convergent extension [40]. These summarized findings support the notion that the PTK7 population is representative for human cells fated to undergo developmental EMT or even gastrulation.

### Transcriptional Profiling of Definitive Endoderm to Contrast with PTK7+ Cells

Because the PTK7 positive population appeared to share characteristics with mesendodermal progenitors such as the expression of SLUG and EOMES, we sought to isolate and profile a distinct developmental stage to compare and distinguish these early developmental decisions. Undifferentiated hESCs were exposed to Activin A, a TGFβ agonist (as described previously) [41], [42], and after 5 days of specification at least 90% of the cells in culture expressed definitive endoderm markers SOX17 and FOXA2 (termed DE hereafter). We normalized these transcriptional profiles and studied the changes in pluripotency, ectoderm, endoderm, and mesoderm markers (Fig. S8).

**Figure 5:**
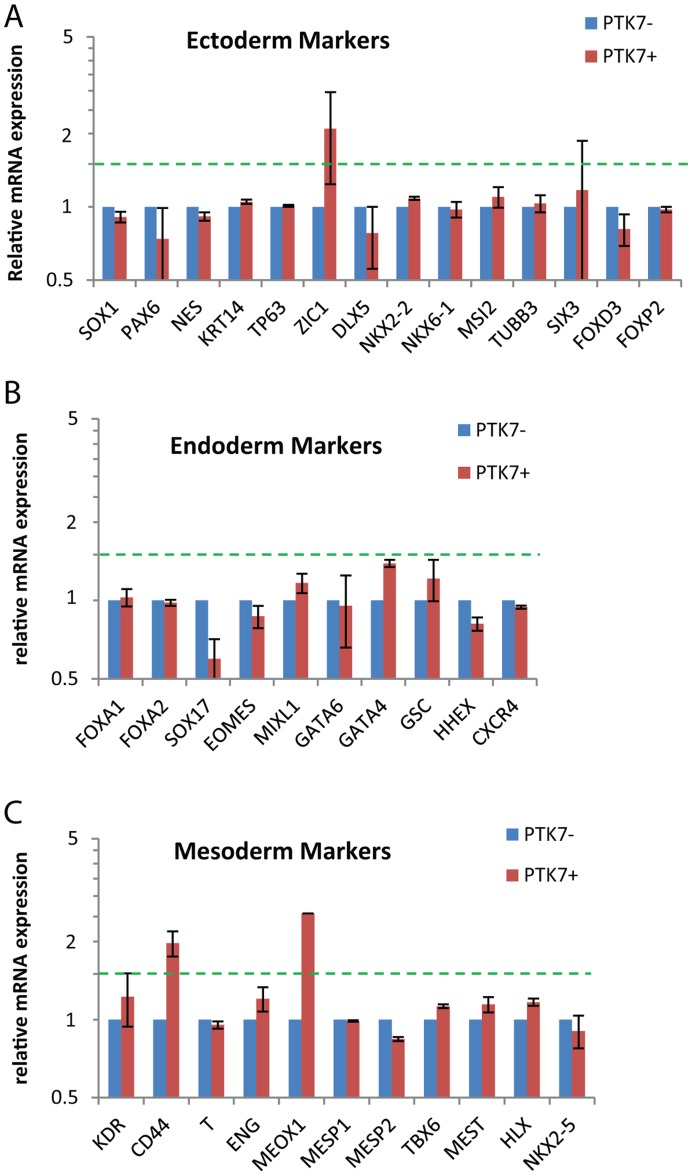
Lineage marker analysis on PTK7− and PTK7+ populations. Feeder-free H9 and XFiPSC2 (differentiated for 2 days) were sorted into PTK7+ and PTK7**−** populations. Expression level was determined by microarray analysis as described in Materials and Methods. All samples were normalized to PTK7**−** from the same sort. Shown are the averages of normalized readings for PTK7+ and PTK7**−**, with standard errors across two experiments. The relative expression of all samples was analyzed for (A) ectoderm markers, (B) endoderm markers and (C) mesoderm markers.

As expected, DE in general did not express increased levels of mesodermal or ectodermal markers (Fig. S8). Among endodermal markers, DE showed significant upregulation of most (*FOXA2, SOX17, EOMES, MIXL1, GATA4, GATA6, GSC* and *CXCR4*) as expected (Fig. S8). In contrast, the PTK7+ population did not show upregulation of these genes (Fig. 5B). These data clearly indicate that the PTK7+ population is distinct from definitive endoderm, despite the fact that these populations can both be generated from hPSCs over a short time course (<3days).

### PTK7+ Population does not Show Changes in Pluripotency and Lineage Markers

Next, we probed if the PTK7+ population presented any changes in pluripotency or lineage markers. The PTK7+ population showed similar levels of pluripotency markers as PTK7**−** cells (Fig. S7A). In addition, the PTK7+ population did not show significant differences from the PTK7**−** counterparts in extra-embryonic (Fig. S7B), ectoderm (Fig. 5A) and endoderm (Fig. 5B) markers. Compared to populations shown to be committed to mesoderm [31], PTK7+ cells only displayed modest upregulation in a handful of these markers (*CD44* at 2.0-fold, *MEOX1* at 2.6-fold), suggesting that this population is likely not yet committed to mesoderm (Fig. 5C).

### Genes Upregulated in PTK7+ Population

We consistently noticed a handful of genes significantly upregulated in PTK7+ populations including: Alpha Smooth Muscle Actin (*α-SMA/ACTA2*, 3.5-fold), Annexin A1 (*ANXA1*, 22.1-fold), Insulin Growth Factor-2 (*IGF2*, 5.8-fold), IGF Binding Protein-7 (*IGFBP7*, 5.7-fold), Rho-related Binding Protein (*RHOJ*, 4.7-fold), Ankyrin Repeat Domain-containing Protein 1 (*ANKRD1*, 6.1-fold), Periostin (*POSTN*, 12.9-fold), Transmembrane 4 L6 Familly Member 1 (*TM4SF1*, 4.7-fold) and Cyclin-dependent Kinase 4 Inhibitor B (*CDKN2B*, 4.8-fold) (Fig. 4C). These genes are not commonly associated with early embryonic development, and they present possible novel candidates for markers or even regulators of early human development.

### Re-aggregation of PTK7+ Population Revealed the Reversible Nature of EMT Marked by PTK7

**Figure 6:**
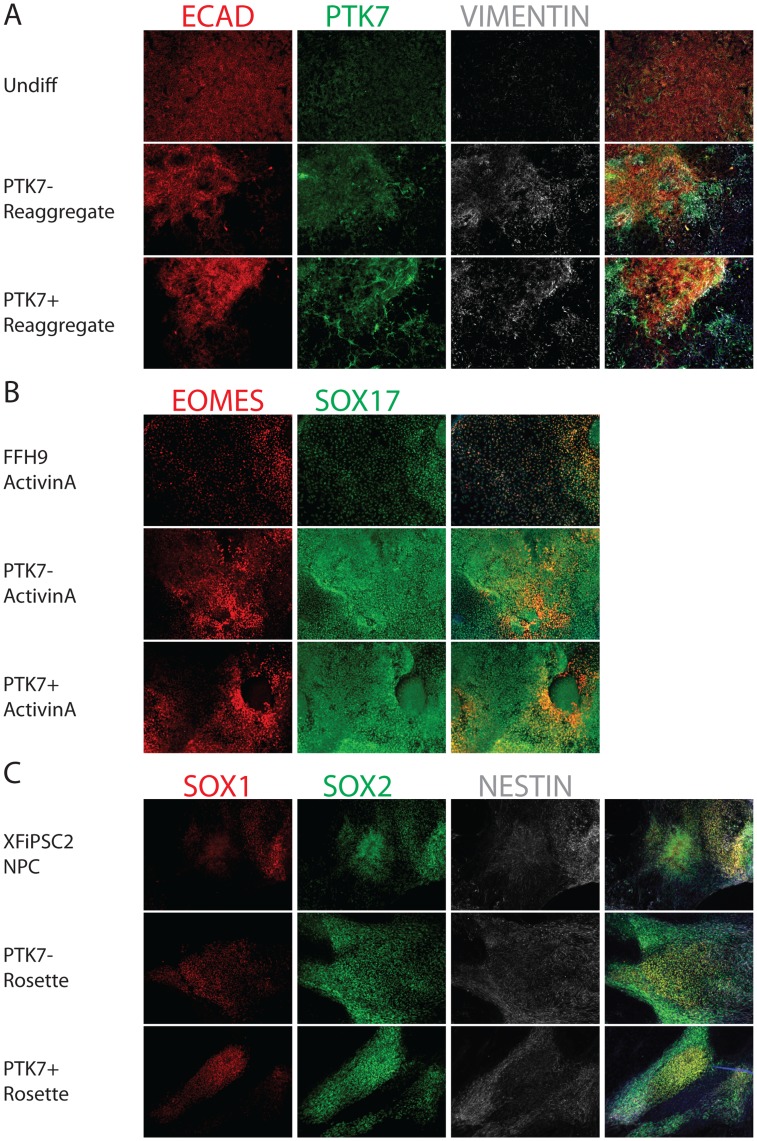
Cell Fate Determination on PTK7− and PTK7+ populations. Feeder-free H9 (differentiated for 2 days) were sorted into PTK7+ and PTK7**−** populations. The two populations were re-aggregated as described in Materials and Methods, and replated onto Matrigel-coated coverslips. The PTK7**−** and PTK7+ re-aggregates were cultured in PSC media for 2 days, and then differentiated using established protocols. (A) Epithelial and mesenchymal marker analysis in PTK7**−** and PTK7+ re-aggregates 3 day post-sorting. PTK7+ cells that underwent developmental EMT possessed plasticity to revert back to an epithelial cell type. Undifferentiated H9 were plated on coverslips and fixed 4 days after passage. PTK7**−** and PTK7+ re-aggregates were plated on coverslips and fixed after 2 days in culture. Undifferentiated H9 (top row), PTK7**−** re-aggregate (mid row) and PTK7+ re-aggregate (bottom row) were stained for PTK7 (green), epithelial maker (E-CADHERIN, red) and mesenchymal marker (VIMENTIN, white). The 4^th^ column shows the merged images from three fluorescent channels and DAPI. (B) PTK7+ and PTK7**−** re-aggregates exhibited comparable definitive endoderm formation. Feeder-free H9, PTK7**−** and PTK7+ re-aggregates were induced to form definitive endoderm with ActivinA treatment as described. Coverslips were fixed and immunostained with endodermal markers EOMES (red) and SOX17 (green). The 3^rd^ column shows the merged images from two fluorescent channels and DAPI. (C) PTK7**−** and PTK7+ re-aggregates exhibited comparable neural differentiation. XFiPSC2, PTK7**−** and PTK7+ re-aggregates were induced to form neural rosettes as previously described. Coverslips were fixed and immunostained with neural markers SOX1 (red), SOX2 (green) and NESTIN (white). The 4^th^ column shows the merged images from three fluorescent channels and DAPI. All images taken at 10X.

In total, the above data suggested that the PTK7+ population could represent cells of the epiblast, proceeding through an EMT on the way to generating mesendodermal lineages. To determine whether the EMT observed in embryoid bodies is irreversible, PTK7+ and PTK7**−** cells were isolated by FACS and re-plated. These isolates were subsequently placed into differentiation conditions towards ectodermal or endodermal lineages. Surprisingly, once either the PTK7+ or PTK7**−** populations were re-plated onto culture dishes, both strongly expressed *E-CA*D and *OCT4* suggesting that the PTK7+ cells could be forced back to an undifferentiated state by simply plating them in PSC culture conditions (Fig. 6A, Fig. S9). Directed differentiation subsequent to re-plating showed that both PTK7+ and PTK7**−** populations were able to generate significant quantities of both ectodermal and endodermal derivatives, suggesting that the EMT observed in EBs was either transient or reversible (Fig. 6B,C).

## Discussion

Embryoid bodies have long been a fascinating research tool since the discovery of teratocarcinoma cell lines. *A priori*, we presumed that human EBs followed a spontaneous random differentiation into three specific germ layers in perhaps a dispersed fashion, as has been described previously [7], [8]. Here we demonstrated that PTK7, a planar cell polarity regulator, is expressed in a population of cells that exhibits characteristics resembling epithelial-to-mesenchymal transition, and that this cell fate decision occurs in groups of cells simultaneously. Although PTK7 was induced just prior to the loss of epithelial nature, the EMT only appeared to proceed when a three-dimensional structure formed on culture plates, or when embryoid bodies were aggregated. While most human studies currently focus on differentiation in terms of growth factor induction and gene expression initiation, regulation of polarity and polarity genes has been shown to play important roles in murine embryonic development. Future work will be required to ascertain the contribution of shape and force in cell fate decisions in this context.

### Probing PTK7+ FACS Isolate for Novel Markers/Regulators in Early Human Development

The PTK7+ population consistently upregulated a handful of genes that are not widely regarded to play roles in early embryonic development. These include genes that are associated with cardiac/muscle development (*α-SMA/ACTA2, ANKRD1)* and bone development (*POSTN*). These genes may act as markers or regulators in an early developmental context. In particular, *ANKRD1* may be an interesting candidate because it has been shown to function as a transcription co-repressor in response to mechanical stress [43], [44]. This is of interest because cell polarity and morphological changes accompany embryonic EMT. The genes upregulated in PTK7+ cells also include those related to cell polarity and motility, such as *RHOJ* and *TM4SF1* [45], [46], [47], [48], [49], [50], along with ECM proteins. In addition to these, IGF signaling pathway players (*IGF2, IGFBP7*) are also among these upregulated genes, possibly suggesting a role of IGF signaling in initiating or aiding the early embryonic EMT. Future studies will focus on manipulating the morphological and polarity changes in embryoid bodies and studying various functional and transcriptional responses.

### PTK7+ Population Shows Upregulation of EMT Markers, but not Mesoderm or Definitive Endoderm Markers

PTK7 positive cells clearly displayed an upregulation of a panel of EMT/primitive streak markers such as *TWIST1*, *SLUG*, *ZEB1*, *FOXC2* and *MMP2*. It is plausible that this EMT event is still at an early stage based on two observations: (1) some of the classic EMT markers, such as *SNAIL1*, are not turned on; (2) in some of the PTK7 positive cells in adherent culture differentiation, E-CAD is not completely turned off, both at the RNA and protein levels. It is likely that the incomplete silencing of *E-CAD* transcription stems from the lack of induction of *SNAIL1*, a well-established inhibitor of *E-CAD* transcription. It is possible that primitive streak formation/gastrulation is initiated by the induction of the genes associated with EMT that we uncovered in the PTK7 positive population, whereas the induction of *SNAIL1* (by growth factors we did not provide in our spontaneous differentiation) completes the transcriptional silencing of *E-CAD* and drives full epithelial-to-mesenchymal transition. The most upregulated genes in the PTK7+ population included *LUMICAN, FIBRONECTIN, DECORIN, TENASIN-C* as well as a vast collection of *COLLAGEN* isotypes, indicating that early remodeling of extra-cellular matrix may pave the way for large-scale tissue patterning and cell migration in human embryonic EMT. This result is intriguing in light of recent work demonstrating that expression of Fibronectin is required for left/right asymmetry in the earliest stages of *Xenopus* development [39].

Also corroborative with the notion that the PTK7 positive population resembles an early developmental EMT is the lack of appreciable induction of definitive mesdoermal and endodermal markers, (Fig. 5B,C) and [31], [32]. It is likely that the PTK7 positive population is not yet committed to mesodermal or endodermal lineages, as we have seen expression of mesodermal markers (*SLUG*) and endodermal markers (*EOMES*) in PTK7 positive population, albeit in a mutually exclusive fashion (Fig. 2J,K). Finally, this system provides an ideal model to study not only EMT, but perhaps also MET, the reverse process, as it appears to occur once PTK7+ cells are re-plated onto plastic culture dishes.

### Conclusion: Impact of Findings on Human Embryology

This work has shed light on the earliest decisions during early human development and describes tools that will open new avenues for future mechanistic discoveries. The isolation of PTK7 positive and negative cells allows us to separate two populations with distinct developmental characteristics. The analysis of the transcriptional profile revealed some expected markers as well as a panel of potentially novel markers to be explored. Our approach has demonstrated, in principle, the potential of pluripotent stem cells and embryoid bodies in modeling the earliest stages of human development.

## Supporting Information

Figure S1 **PTK7+ population displays upregulation of mesenchymal markers and loss of pluripotency markers in hEBs from Day0 to Day4.** Immunofluorescence staining of HSF1 hEB serial sections. HSF1 hEB were cultured for 0, 1, 2, 4, 7, or 10 days before cryosectioned. Cryosections of HSF1 hEB were co-stained with PTK7 (green), mesenchymal marker (VIMENTIN, white), and pluripotency marker (NANOG, red). The 4^th^ column shows the merged images from three fluorescent channels and DAPI. Sections employed in this figure are serial to corresponding ones in Figure S2 and Figure S3.
(TIF)

Figure S2 **PTK7+ population displays upregulation of mesenchymal markers and loss of epithelial markers in hEBs from Day0 to Day4.** Immunofluorescence staining of HSF1 hEB serial sections. HSF1 hEB were cultured for 0, 1, 2, 4, 7,or 10 days before cryosectioned. Cryosections of HSF1 hEB were co-stained with PTK7 (green), mesenchymal marker (VIMENTIN, white), and epithelial marker (E-CAD, red). The 4^th^ column shows the merged images from three fluorescent channels and DAPI. Sections employed in this figure are serial to corresponding ones in Figure S1 and Figure S3.
(TIF)

Figure S3 **PTK7+ population displays upregulation of mesenchymal markers in hEBs from Day0 to Day4.** Immunofluorescence staining of HSF1 hEB serial sections. HSF1 hEB were cultured for 0, 1, 2, 4, 7,or 10 days before cryosectioned. Cryosections of HSF1 hEB were co-stained with PTK7 (green) and mesenchymal markers (N-CAD, red; VIMENTIN, white). The 4^th^ column shows the merged images from three fluorescent channels and DAPI. Sections employed in this figure are serial to corresponding ones in Figure S1 and Figure S2.
(TIF)

Figure S4 **Pluripotency, lineage, and viability marker analysis of PTK7 population in hEBs.** Immunofluorescence staining of XFiPSC2 hEB serial sections. White boundary indicates the PTK7 population. In cases where there were no PTK7 staining in a particular section, the boundary was extrapolated from adjacent sections. XFiPSC2 hEB were cultured for 24 hrs before cryosectioned. Serial sections of XFiPSC2 24 hr hEB are stained with PTK7 (green), epithelial markers (E-CADHERIN, red; EPCAM, green), mesenchymal markers (N-CADHERIN, red; VIMENTIN, white), pluripotency markers (OCT4, SOX2, NANOG, red), developmental genes (LIN28A, LIN28B, HMGA2, red), primitive streak markers (SLUG, red; Brachyury, white) and endodermal markers (SOX17, green; EOMES, red; FOXA2, white). The 4^th^ column shows the merged images from three fluorescent channels and DAPI.
(TIF)

Figure S5 **Quantification of Ki-67+ and Casp3+ cells in PTK7+ and PTK7− populations.** From 24 hr XFiPSC hEB sections, we quantified the proportions of Ki-67 and Caspase3 positive cells in PTK7+ and PTK7**−** populations. The table listed averaged percentages over hEBs quantified from 3 representative images, with standard errors.
(PDF)

Figure S6 **PTK7 Sorting of hEBs.** (A) FACS-plot of PTK7 Sorting. Shown is the FACS plot for sorting of differentiating H9 culture. Left panel showed the FACS-plot of a secondary antibody only control, right panel showed the fully stained sample. From the stained sample, P5 was captured as the PTK7+ population and P6 was captured as the PTK7**−** population. (B) PTK7**−**sorted hEBs displayed upregulation of *PTK7*, *N-CAD* and downregulation of *E-CAD*. XFiPSC2 EBs were sorted into PTK7+ and PTK7**−** population. Reverse-transcription PCR was performed. mRNA expression of PTK7, E-CADHERIN and N-CADHERIN was normalized to that of GAPDH. Error bars represent the standard error over four technical replicates. Results are representative of two independent rtPCR experiments.
(PDF)

Figure S7 **Pluripotency and extraembryonic marker analysis on PTK7− and PTK7+ populations.** Feeder-free H9 and XFiPSC2 (differentiated for 2 days) were sorted into PTK7+ and PTK7**−** populations. Expression level was determined by microarray analysis as described in Materials and Methods. All samples were normalized to PTK7**−** from the same sort. Shown are the averages of normalized readings for PTK7+ and PTK7**−**, with standard errors across two experiments. The relative expression of all samples was analyzed for (A) pluripotency markers and (B) extraembryonic markers.
(PDF)

Figure S8 **Lineage marker analysis on undifferentiated PSC and DE populations.** Definitive endoderm was induced by treating HSF1 with Activin A for 5 days (HSF1 DE). Expression level was determined by microarray analysis as described in Materials and Methods. All samples were normalized to undifferentiated HSF1 (HSF1 Undiff). The relative expression of all samples was analyzed for (A) ectoderm markers, (B) endoderm markers, (C) mesoderm markers and (D) pluripotency markers.
(PDF)

Figure S9 **Pluripotency Marker Expression in PTK7− and PTK7+ re-aggregates.** Feeder-free H9 (differentiated for 2 days) were sorted into PTK7+ and PTK7**−** populations. The two populations were re-aggregated as described in Materials and Methods, and replated onto Matrigel-coated coverslips. The PTK7**−** and PTK7+ re-aggregates were cultured in PSC media for 2 days. Pluripotency marker was analyzed in PTK7**−** and PTK7+ re-aggregates 3 day post-sorting. PTK7+ cells that underwent developmental EMT possessed plasticity to regain pluripotency marker OCT4. Undifferentiated H9 were plated on coverslips and fixed 4 days after passage. PTK7**−** and PTK7+ re-aggregates were plated on coverslips and fixed after 2 days in culture. Undifferentiated H9 (top row), PTK7**−** re-aggregate (mid row) and PTK7+ re-aggregate (bottom row) were stained for N-CAD(red), pluripotency maker (OCT4, green) and mesenchymal marker (VIMENTIN, white). The 4^th^ column shows the merged images from three fluorescent channels and DAPI. All images taken at 10X.
(TIF)
