# Supplementary figures and images for: Ptk7 Marks the First Human Developmental EMT In Vitro
Source: PLoS One. 2012 Nov 28;7(11):e50432. doi: 10.1371/journal.pone.0050432 (PMC3508926; doi:10.1371/journal.pone.0050432)

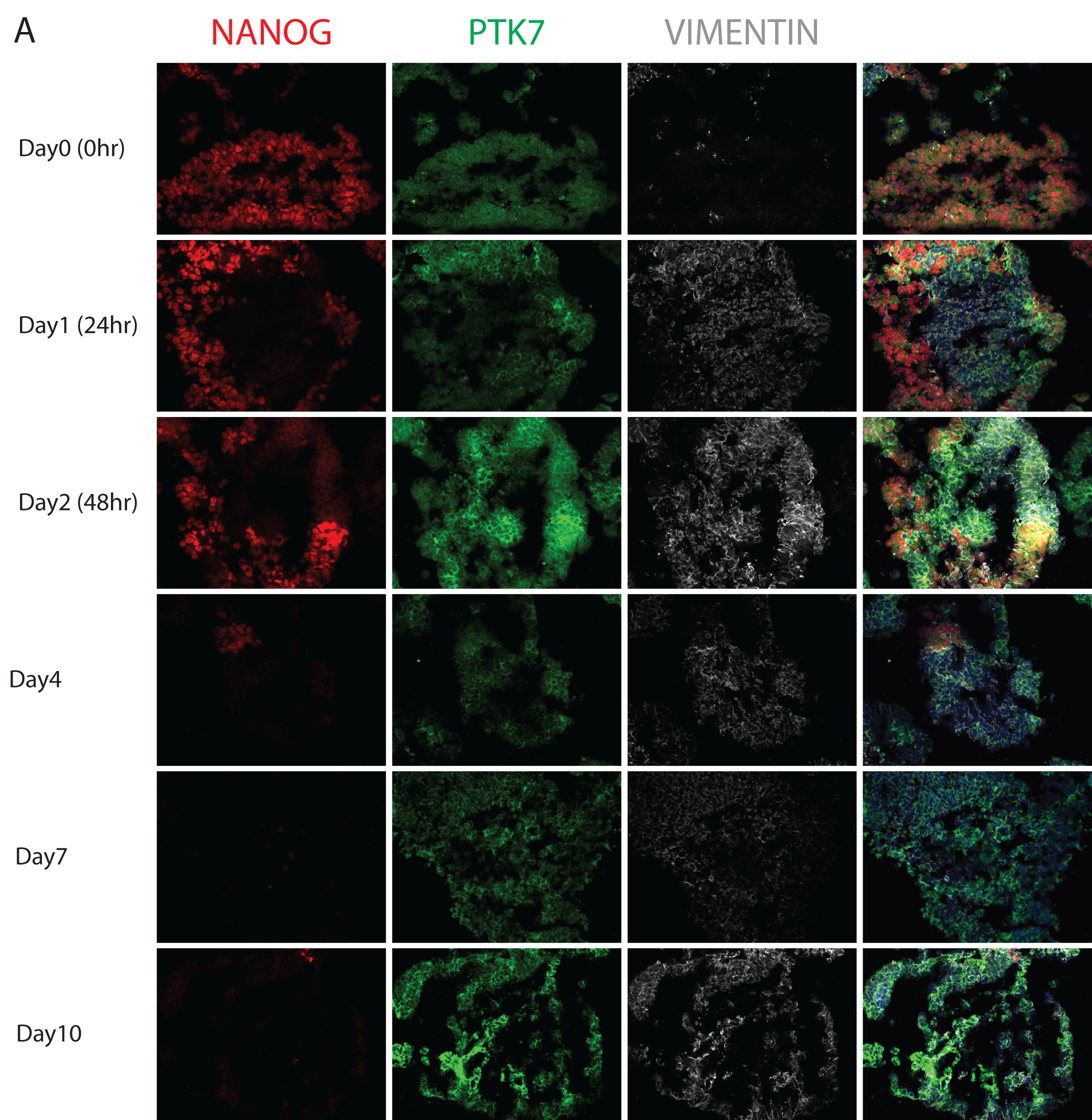

Supplement: Figure S1 — PTK7+ population displays upregulation of mesenchymal markers and loss of pluripotency markers in hEBs from Day0 to Day4. Immunofluorescence staining of HSF1 hEB serial sections. HSF1 hEB were cultured for 0, 1, 2, 4, 7, or 10 days before cryosectioned. Cryosections of HSF1 hEB were co-stained with PTK7 (green), mesenchymal marker (VIMENTIN, white), and pluripotency marker (NANOG, red). The 4th column shows the merged images from three fluorescent channels and DAPI. Sections employed in this figure are serial to corresponding ones in Figure S2 and Figure S3. (TIF) [file pone.0050432.s001.tif]

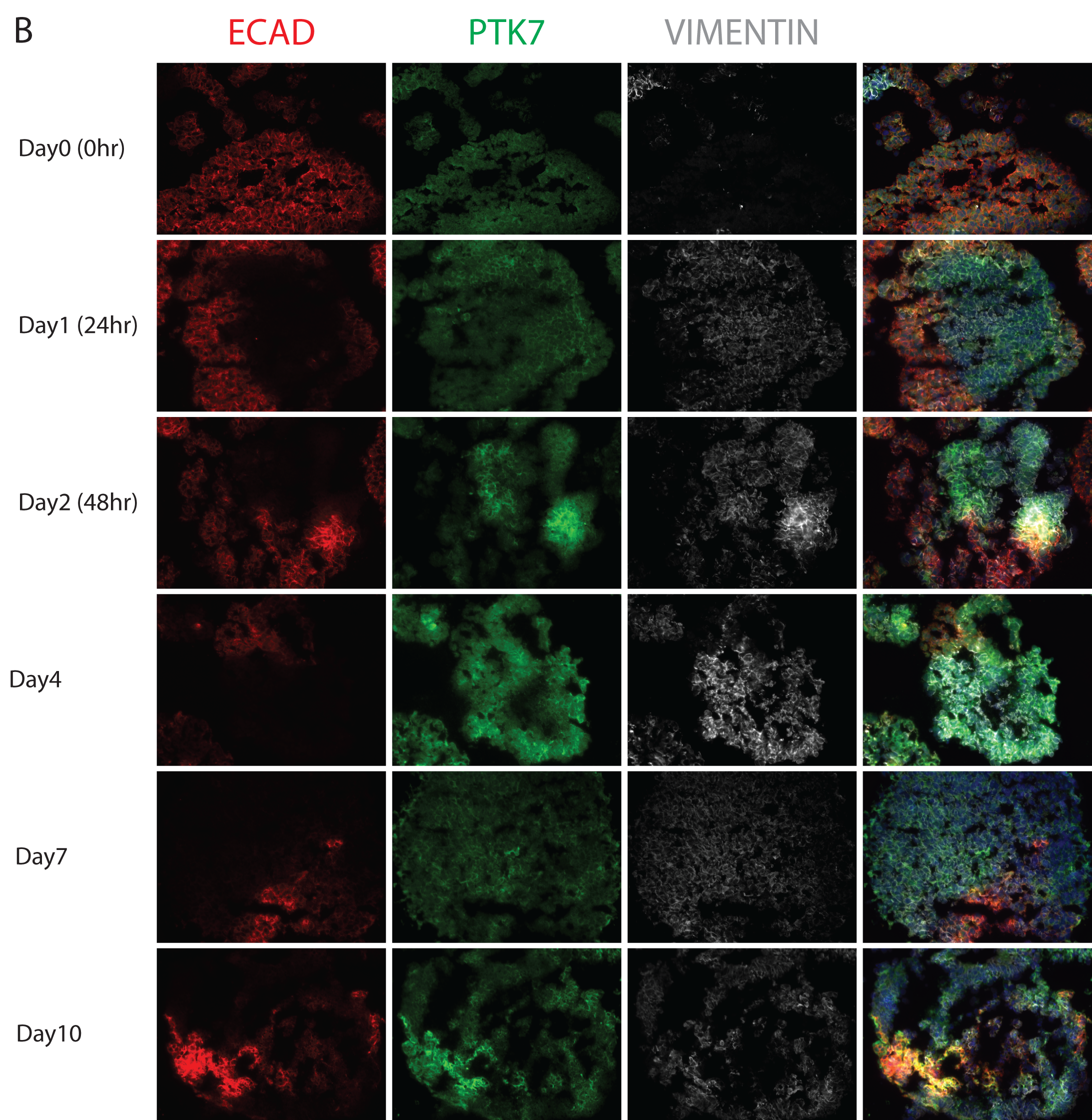

Supplement: Figure S2 — PTK7+ population displays upregulation of mesenchymal markers and loss of epithelial markers in hEBs from Day0 to Day4. Immunofluorescence staining of HSF1 hEB serial sections. HSF1 hEB were cultured for 0, 1, 2, 4, 7,or 10 days before cryosectioned. Cryosections of HSF1 hEB were co-stained with PTK7 (green), mesenchymal marker (VIMENTIN, white), and epithelial marker (E-CAD, red). The 4th column shows the merged images from three fluorescent channels and DAPI. Sections employed in this figure are serial to corresponding ones in Figure S1 and Figure S3. (TIF) [file pone.0050432.s002.tif]

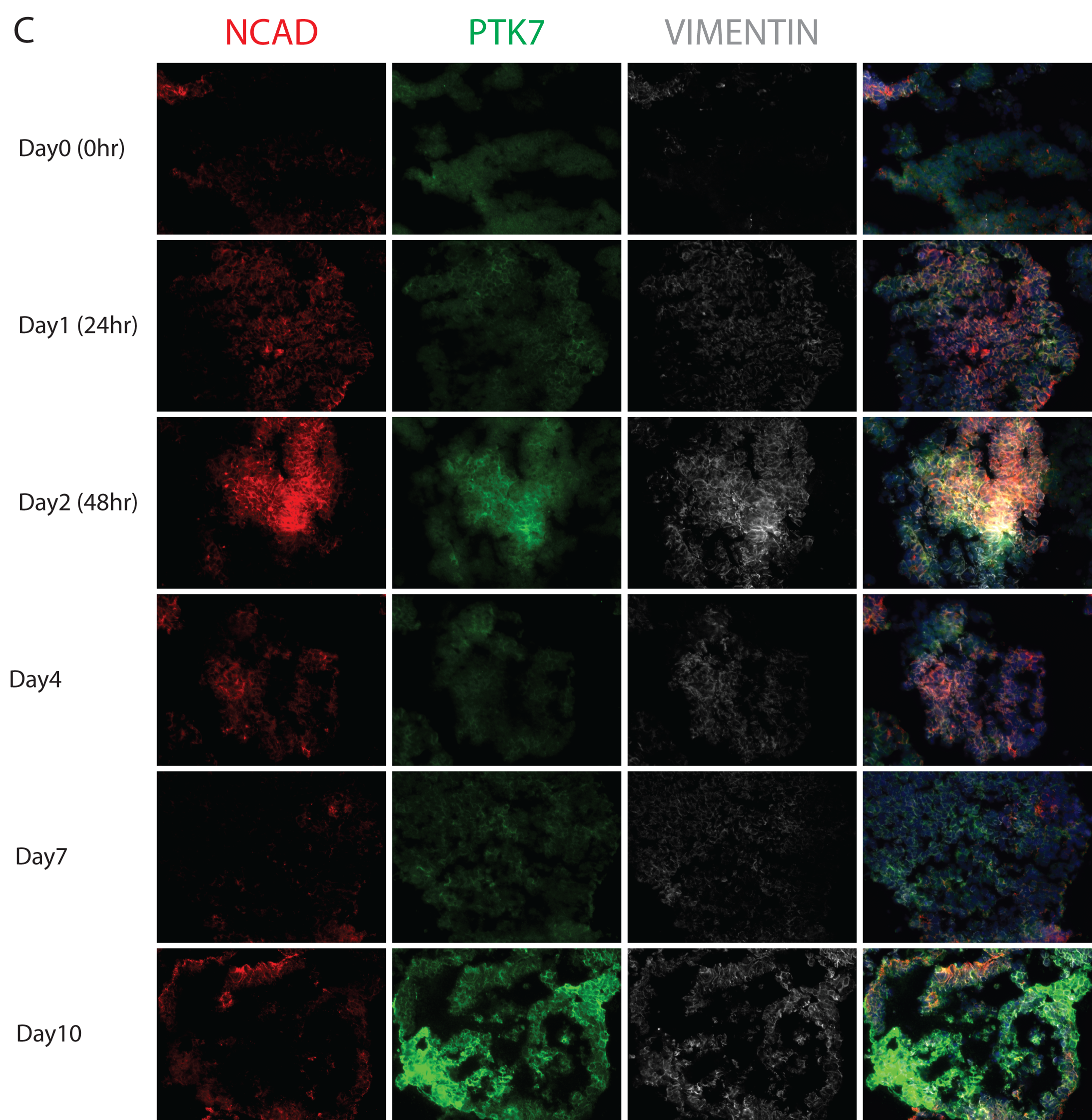

Supplement: Figure S3 — PTK7+ population displays upregulation of mesenchymal markers in hEBs from Day0 to Day4. Immunofluorescence staining of HSF1 hEB serial sections. HSF1 hEB were cultured for 0, 1, 2, 4, 7,or 10 days before cryosectioned. Cryosections of HSF1 hEB were co-stained with PTK7 (green) and mesenchymal markers (N-CAD, red; VIMENTIN, white). The 4th column shows the merged images from three fluorescent channels and DAPI. Sections employed in this figure are serial to corresponding ones in Figure S1 and Figure S2. (TIF) [file pone.0050432.s003.tif]

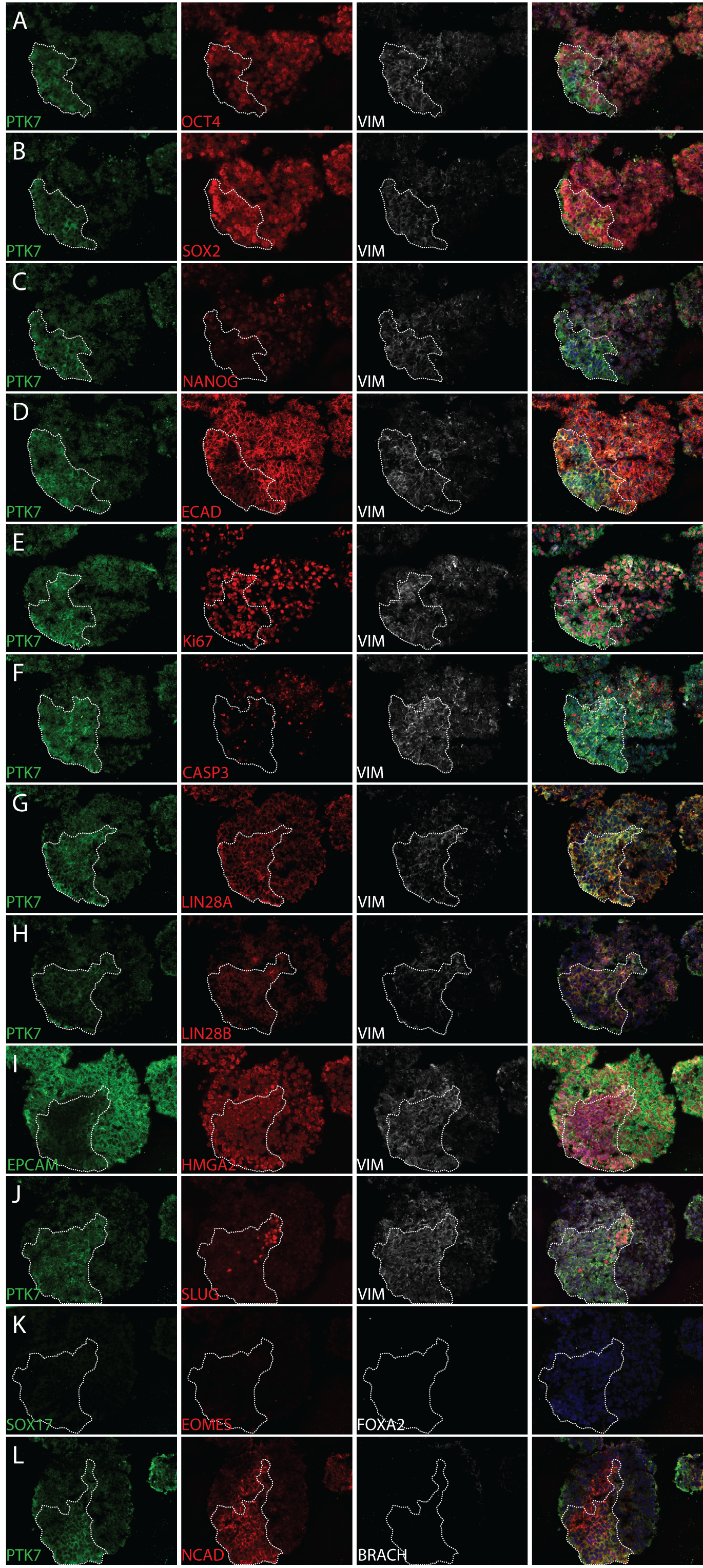

Supplement: Figure S4 — Pluripotency, lineage, and viability marker analysis of PTK7 population in hEBs. Immunofluorescence staining of XFiPSC2 hEB serial sections. White boundary indicates the PTK7 population. In cases where there were no PTK7 staining in a particular section, the boundary was extrapolated from adjacent sections. XFiPSC2 hEB were cultured for 24 hrs before cryosectioned. Serial sections of XFiPSC2 24 hr hEB are stained with PTK7 (green), epithelial markers (E-CADHERIN, red; EPCAM, green), mesenchymal markers (N-CADHERIN, red; VIMENTIN, white), pluripotency markers (OCT4, SOX2, NANOG, red), developmental genes (LIN28A, LIN28B, HMGA2, red), primitive streak markers (SLUG, red; Brachyury, white) and endodermal markers (SOX17, green; EOMES, red; FOXA2, white). The 4th column shows the merged images from three fluorescent channels and DAPI. (TIF) [file pone.0050432.s004.tif]

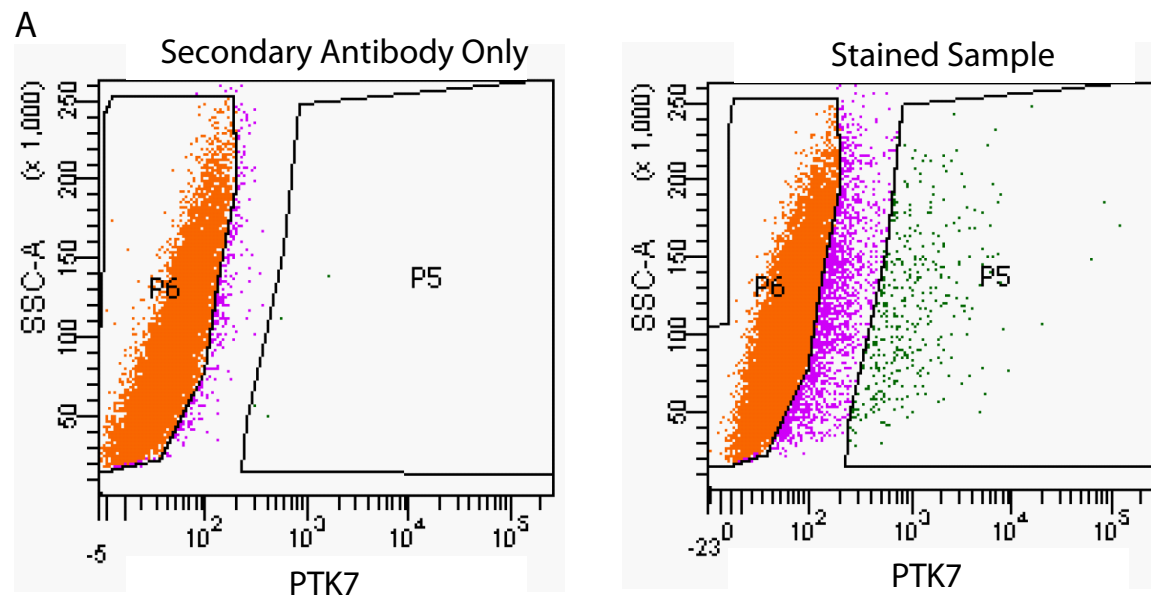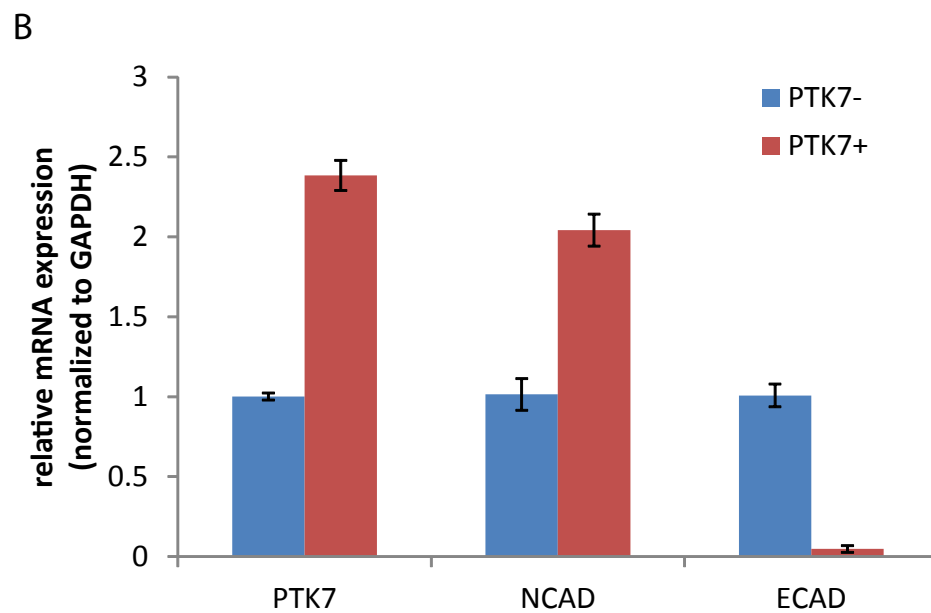

Supplement: Figure S6 — PTK7 Sorting of hEBs. (A) FACS-plot of PTK7 Sorting. Shown is the FACS plot for sorting of differentiating H9 culture. Left panel showed the FACS-plot of a secondary antibody only control, right panel showed the fully stained sample. From the stained sample, P5 was captured as the PTK7+ population and P6 was captured as the PTK7− population. (B) PTK7−sorted hEBs displayed upregulation of PTK7, N-CAD and downregulation of E-CAD. XFiPSC2 EBs were sorted into PTK7+ and PTK7− population. Reverse-transcription PCR was performed. mRNA expression of PTK7, E-CADHERIN and N-CADHERIN was normalized to that of GAPDH. Error bars represent the standard error over four technical replicates. Results are representative of two independent rtPCR experiments. (PDF) [file pone.0050432.s006.pdf]

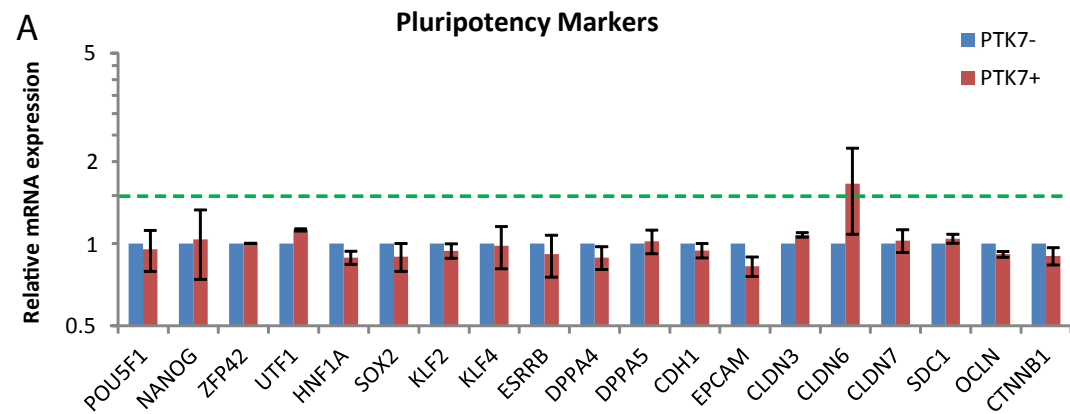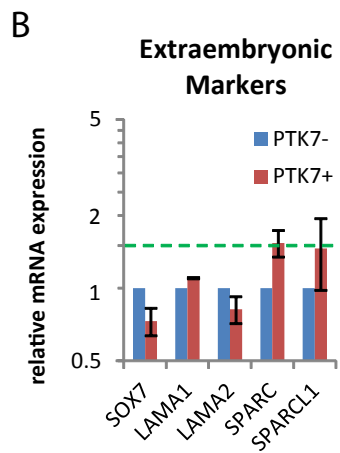

Supplement: Figure S7 — Pluripotency and extraembryonic marker analysis on PTK7− and PTK7+ populations. Feeder-free H9 and XFiPSC2 (differentiated for 2 days) were sorted into PTK7+ and PTK7− populations. Expression level was determined by microarray analysis as described in Materials and Methods. All samples were normalized to PTK7− from the same sort. Shown are the averages of normalized readings for PTK7+ and PTK7−, with standard errors across two experiments. The relative expression of all samples was analyzed for (A) pluripotency markers and (B) extraembryonic markers. (PDF) [file pone.0050432.s007.pdf]

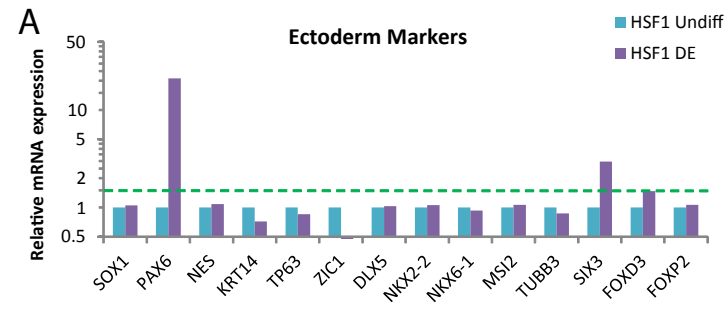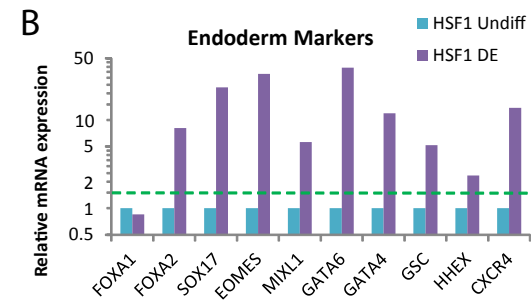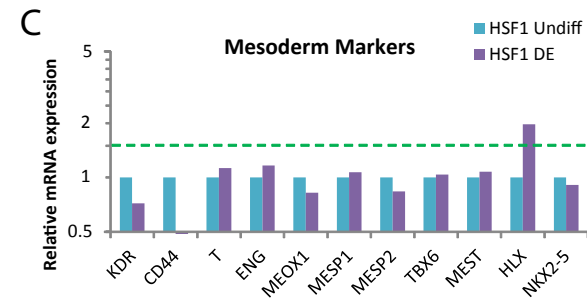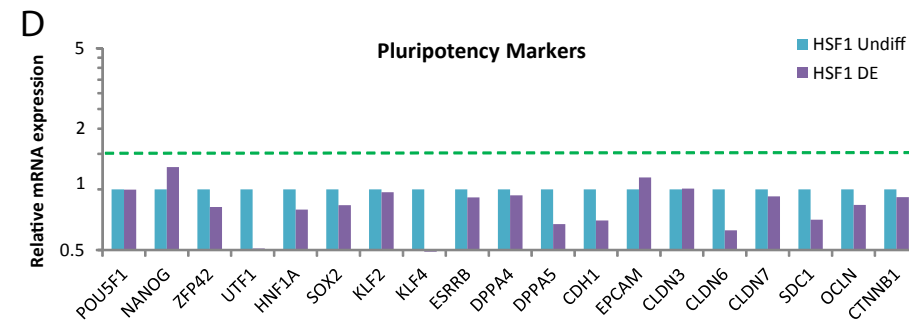

Supplement: Figure S8 — Lineage marker analysis on undifferentiated PSC and DE populations. Definitive endoderm was induced by treating HSF1 with Activin A for 5 days (HSF1 DE). Expression level was determined by microarray analysis as described in Materials and Methods. All samples were normalized to undifferentiated HSF1 (HSF1 Undiff). The relative expression of all samples was analyzed for (A) ectoderm markers, (B) endoderm markers, (C) mesoderm markers and (D) pluripotency markers. (PDF) [file pone.0050432.s008.pdf]

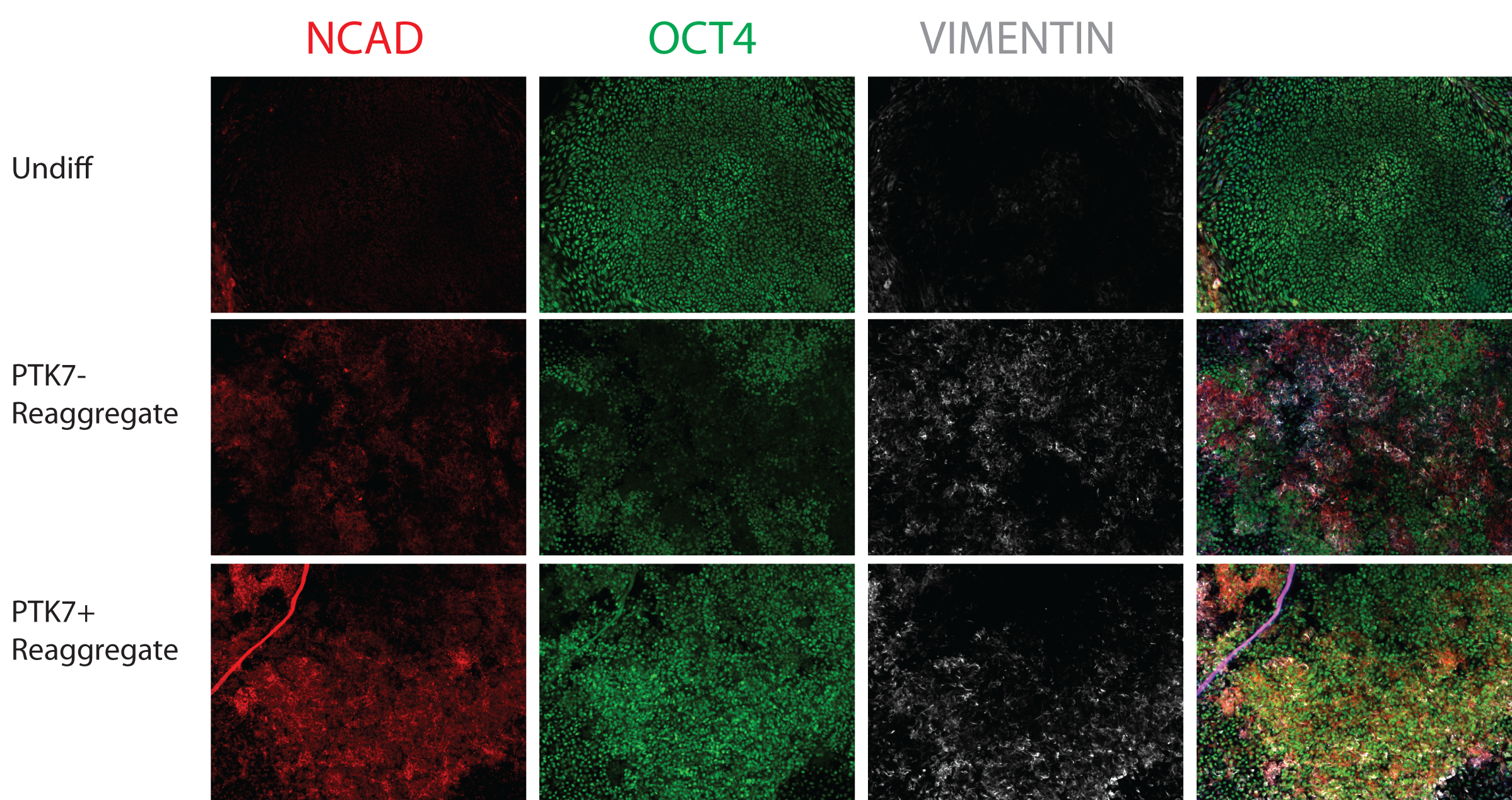

Supplement: Figure S9 — Pluripotency Marker Expression in PTK7− and PTK7+ re-aggregates. Feeder-free H9 (differentiated for 2 days) were sorted into PTK7+ and PTK7− populations. The two populations were re-aggregated as described in Materials and Methods, and replated onto Matrigel-coated coverslips. The PTK7− and PTK7+ re-aggregates were cultured in PSC media for 2 days. Pluripotency marker was analyzed in PTK7− and PTK7+ re-aggregates 3 day post-sorting. PTK7+ cells that underwent developmental EMT possessed plasticity to regain pluripotency marker OCT4. Undifferentiated H9 were plated on coverslips and fixed 4 days after passage. PTK7− and PTK7+ re-aggregates were plated on coverslips and fixed after 2 days in culture. Undifferentiated H9 (top row), PTK7− re-aggregate (mid row) and PTK7+ re-aggregate (bottom row) were stained for N-CAD(red), pluripotency maker (OCT4, green) and mesenchymal marker (VIMENTIN, white). The 4th column shows the merged images from three fluorescent channels and DAPI. All images taken at 10X. (TIF) [file pone.0050432.s009.tif]
