# Supplementary material for: Ptk7 Marks the First Human Developmental EMT In Vitro
Source: PLoS One. 2012 Nov 28;7(11):e50432. doi: 10.1371/journal.pone.0050432 (PMC3508926; doi:10.1371/journal.pone.0050432)
Supplement: Figure S5 — Quantification of Ki-67+ and Casp3+ cells in PTK7+ and PTK7− populations. From 24 hr XFiPSC hEB sections, we quantified the proportions of Ki-67 and Caspase3 positive cells in PTK7+ and PTK7− populations. The table listed averaged percentages over hEBs quantified from 3 representative images, with standard errors. (PDF) [file pone.0050432.s005.pdf]

**hEB population**	**% Ki67 positive**	**% Caspase3 positive**
PTK7+	76.1% ± 2.8%	3.1% ± 1.6%
PTK7-	69.3% ± 4.9%	15.8% ± 2.5%
